# Immune Response and Mitochondrial Metabolism Are Commonly Deregulated in DMD and Aging Skeletal Muscle

**DOI:** 10.1371/journal.pone.0026952

**Published:** 2011-11-09

**Authors:** Daniel Baron, Armelle Magot, Gérard Ramstein, Marja Steenman, Guillemette Fayet, Catherine Chevalier, Philippe Jourdon, Rémi Houlgatte, Frédérique Savagner, Yann Pereon

**Affiliations:** 1 INSERM, UMR915, Nantes, France; 2 Université de Nantes, Nantes, France; 3 L'Institut du Thorax, CHU de Nantes, CIC, Nantes, France; 4 Laboratoire d'Explorations Fonctionnelles, CHU de Nantes, Nantes, France; 5 Centre de Référence des Maladies Neuromusculaires Rares de l'Enfant et de l'Adulte Nantes-Angers, CHU de Nantes, Nantes, France; 6 Laboratoire d'Informatique de Nantes Atlantique LINA, Ecole Polytechnique, Nantes, France; 7 INSERM, UMR 694, Angers, France; 8 Université d'Angers, Angers, France; Saint Louis University, United States of America

## Abstract

Duchenne Muscular Dystrophy (DMD) is a complex process involving multiple pathways downstream of the primary genetic insult leading to fatal muscle degeneration. Aging muscle is a multifactorial neuromuscular process characterized by impaired muscle regeneration leading to progressive atrophy. We hypothesized that these chronic atrophying situations may share specific myogenic adaptative responses at transcriptional level according to tissue remodeling. Muscle biopsies from four young DMD and four AGED subjects were referred to a group of seven muscle biopsies from young subjects without any neuromuscular disorder and explored through a dedicated expression microarray. We identified 528 differentially expressed genes (out of 2,745 analyzed), of which 328 could be validated by an exhaustive meta-analysis of public microarray datasets referring to DMD and Aging in skeletal muscle. Among the 328 validated co-expressed genes, 50% had the same expression profile in both groups and corresponded to immune/fibrosis responses and mitochondrial metabolism. Generalizing these observed meta-signatures with large compendia of public datasets reinforced our results as they could be also identified in other pathological processes and in diverse physiological conditions. Focusing on the common gene signatures in these two atrophying conditions, we observed enrichment in motifs for candidate transcription factors that may coordinate either the immune/fibrosis responses (ETS1, IRF1, NF1) or the mitochondrial metabolism (ESRRA). Deregulation in their expression could be responsible, at least in part, for the same transcriptome changes initiating the chronic muscle atrophy. This study suggests that distinct pathophysiological processes may share common gene responses and pathways related to specific transcription factors.

## Introduction

Skeletal muscles comprise 40–50% of the total adult human body mass and are responsible for a number of functions including force generation, movement and body support. Skeletal muscle size depends upon a dynamic balance between hypertrophic and atrophic processes. The maintenance of muscle fiber size is tightly regulated by intracellular signaling networks that determine the balance between overall rates of protein synthesis and degradation [1]. While muscle hypertrophy occurs with physical exercise [2], there are a number of physiological or pathological conditions in which a loss of skeletal muscle mass occurs [3]. They include Duchenne muscular dystrophy (DMD) which is characterized by rapidly progressive muscle degeneration primary caused by lack of dystrophin, and sarcopenia which specifically refers to the irreversible decline of skeletal muscle mass, strength, and function with age [4].

Dystrophin is a large protein located at the intracellular region of the sarcolemma and participates to cellular adhesion by connecting the cytoskeleton to the extracellular matrix [5]. This supported the mechanical strength of the sarcolemma against muscle contraction-induced tension [6]. It was generally admitted that in muscle lacking dystrophin, this connection was weakened and sarcolemma was exposed to high tension. That led to partial disruptions of sarcolemma and influx of extracellular Ca^2+^ that activated the calcium-dependent degradative pathway, myofibril disruption and muscle necrosis or apoptosis [7] along with incomplete regeneration cycles, proliferation of connective and adipose tissue, infiltration of immune cells and reduced number of slow oxidative muscle fibers [8].

Sarcopenia is associated with a preferential alteration in the number and size of type II muscle fibers. Many neuro-muscular and systemic factors have been implicated in the pathogenesis of sarcopenia including loss of type II alpha motor neurons, decline in muscle fiber contractility, deficient satellite cell recruitment, enhanced oxidative stress, mitochondrial dysfunction, loss of growth and sex hormone production, activation of proteolytic pathway, dysregulation of catabolic cytokines [9]. At the single muscle fiber level, sarcopenia was associated to hallmarks of morphological apoptosis, decline in protein synthesis increased DNA, protein and lipid oxidation, accumulation of mitochondrial abnormalities and calcium dyshomeostasis [10]–[14].

Taken together, these data suggest that common remodeling process occurred in both pathological and aging atrophying conditions and that it may be related to transcriptional alterations affecting numerous molecular pathways and biological functions, modifying tissue and morphological characteristics of the muscle. We hypothesized that common gene signatures may arise in the DMD and aged human skeletal muscle. One tool that might define a molecular portrait of muscle in these conditions is gene expression profiling using microarrays [15]. This method has been successfully applied in the field of muscle research, particularly for several human diseases [16], [17]. We thus performed gene expression profiling in skeletal muscle biopsies from DMD and aged patients. The results, meta-analyzed in the context of available public microarray data, enabled us to highlight common molecular signatures. Here we demonstrated that DMD and AGE share common transcriptional changes related to the tissue remodeling occurring in the atrophying skeletal muscle. Moreover, the observed patterns could be regulated by common transcription factors (TFs) to be identified.

## Results

### In laboratory study

#### Hierarchical clustering of microarray data

RNA samples from 4 DMD and 4 AGE skeletal muscle tissues were analyzed by hybridization against control samples. This hybridization was done on microarrays containing quadruplicate 50-mer oligonucleotides corresponding to 3,588 muscle relevant genes. Two hybridizations were performed for each subject. Thus, eight values per gene were obtained for each subject. These stringent conditions conferred powerful significance to our results.

**Figure 1:**
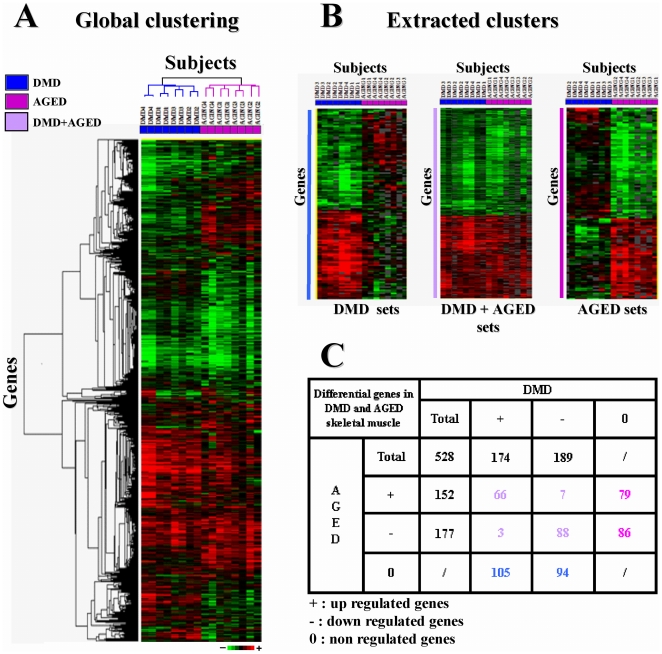
In lab Gene expression profiling in DMD and AGED skeletal muscle. **A-** Two-way hierarchical clustering of expression values measured in our lab from the 8 subjects (4 DMD and 4 AGED) on the 2,745 validated genes (i.e genes having reliable expression values). Each row represents a gene, and each column represents a sample. The dendrogram represents correlation distances between expression levels of genes (horizontally) or samples (vertically). Blue and purple color code is associated to DMD and AGED samples, respectively. Each cell in the matrix corresponds to an expression level, with red for over-expression, green for under-expression, and black for gene expression close to the median (see the color scale). **B-** Three distinct sets of differentially expressed genes (DEGs) have been identified from the 528 significant genes extracted by SAM and LIMMA analysis: (1) DMD set consists in 199 differential genes expression profiles only in DMD (up and down regulated genes) (2) AGED set consists in 165 differential gene expression profiles only in aged (up and down regulated genes). (3) DMD+AGED set consists in 164 common gene expression profiles (up and down regulated genes). **C-** Number of genes associated to each set according to their expression level. Pink values: genes differentially regulated in AGED set only, blue values: genes differentially regulated in DMD set only, purple values: genes differentially regulated in DMD+AGED set only. In DMD set, 105 genes were over expressed and 94 under expressed. In AGED set, 79 genes were over expressed and 86 under expressed. In DMD+AGED set, 66 up regulated and 88 down regulated genes were consistently deregulated, 10 (7 DMD− AGED+; 3 DMD+ AGED−) genes were deregulated in the opposite sense.

After the filtering procedure and the consolidation step, valid expression values (i.e relevant measure of the spot signal above the background) were obtained for 2,745 genes in all subjects. Clustering was performed using the median of the four values per microarray of these 2,745 genes for DMD and AGE subjects (**Figure 1.A**). As expected, duplicate microarrays clustered together and two distinctive clusters appeared: one consisting of DMD patients and one of AGE subjects.Two tests were then performed: DMD vs CONTROL and AGE vs CONTROL. After *s*tatistical analyses (SAM and LIMMA), 528 genes were found to be differentially expressed (p<0.01). Among this list, 3 distinct sets of differentially expressed genes (DEGs) were identified: DMD set (DEGs in DMD but not in aging muscle), AGE set (DEGs in aging muscle but not in DMD muscle), and DMD-AGE set (genes commonly modified in the two situations and corresponding to the overlap between the two tests) (**Figure 1.B**). For each set we found up and down regulated genes as described in **Figure 1.C**. DMD set was composed of 363 genes and AGE set of 329 genes. Majority of genes found in DMD+AGE set were consistently deregulated (66 up-regulated and 88 down regulated in both situations), 10 were inconsistently deregulated (i.e. deregulated in the opposite sense). Complete gene lists are available in **Table S1** (part 1).

#### Quantitative RT-PCR validation

**Figure 2:**
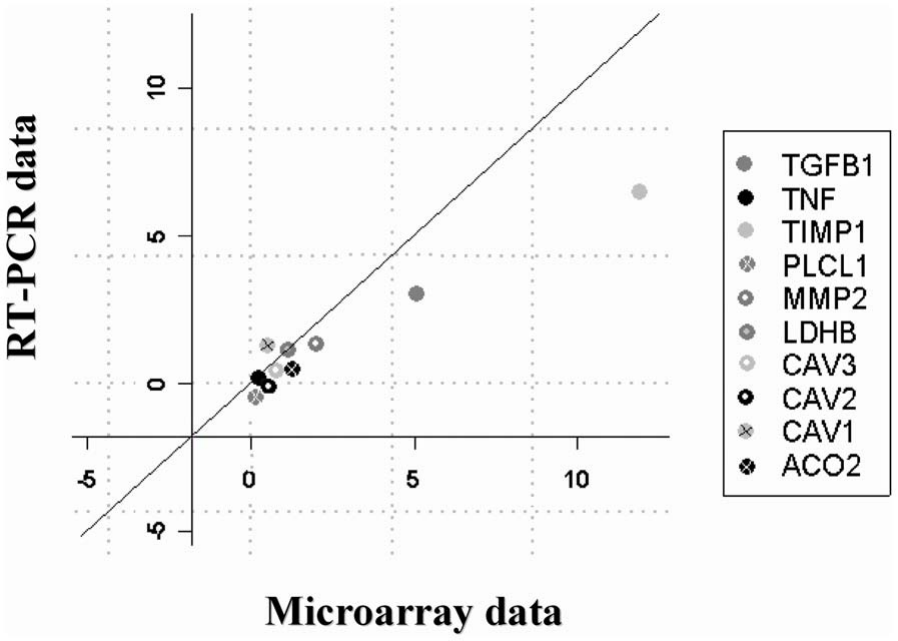
Independent verification of microarray results by quantitative RT-PCR for a group of selected genes in a DMD patient. Scatterplot illustrating the good concordance for 10 gene expression values measured by RT-PCR (y axis) and Microarray (x axis) in one of the DMD samples. Each spot represents mean expression value measured for one gene either by RT-PCR (n = 3) or by microarray experiment. The diagonal line denotes the identity between RT-PCR and microarray expression values. Measured genes are listed in legend. RT PCR expression values are well correlated with microarray expression values (Pearson's correlation r = 0.88).

Differential expression of 10 genes was confirmed by quantitative RT-PCR analysis of 3 of the 4 DMD and 3 of the 4 AGE subjects' biopsies. Ten genes representing different levels of up- or down-regulation in both conditions were tested and HERZ was used as internal control. Figure 2 shows an example of the good correlation between RT-PCR and microarray results for one DMD patient (Pearson's correlation, r = 0.88). Identical correlations were observed for all patients (data not shown) supporting technical validation of the microarray results.

### Orientated Validation: data processing from DMD and AGE public studies

**Figure 3:**
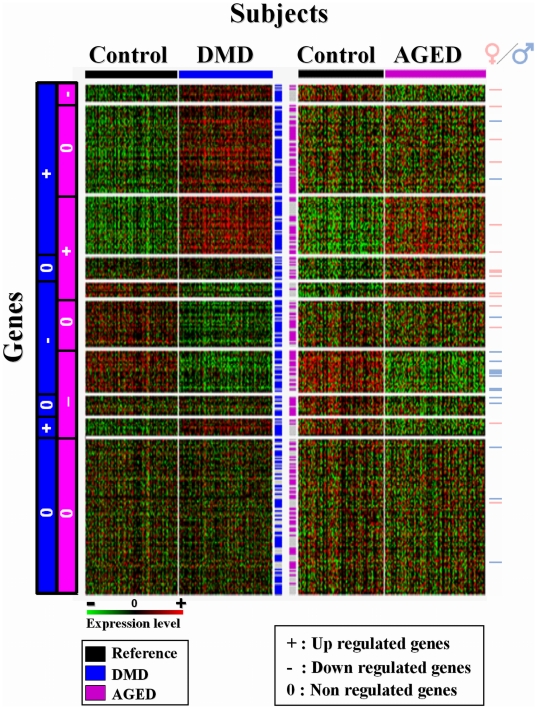
Meta-clusters specifically extracted from independent DMD and AGED public studies. Individualization of 10 metaclusters by two-way hierarchical clustering of expression values across 6 DMD and 6 AGED public microarray studies. Each row represents a gene, and each column represents a subject found in either DMD or AGED studies. Blue and purple color code shows DMD and AGED samples respectively. Each cell in the matrix corresponds to an expression level, with red for over-expression, green for under-expression, and black for gene expression close to the median (see the color scale). We identified genes modified either in at least DMD or AGED, commonly modified in both situations or not modified. Genes cross-validated either in DMD studies either in Aging studies are showed in the center of the matrix with blue and purple pixels respectively. Genes having significant sex biased expression are indicated with pixels (pink for higher expression in female muscle; light blue for higher expression in male muscle) on the right of the matrix.

To validate our microarray results, we performed a meta-analysis on the genes we found differentially expressed in at least one of the two conditions (DMD or AGE) (**Figure 3**). We validated 233 common genes across 6 distinct DMD data sets, and 178 genes across 6 distinct AGE data sets. Those DMD and AGE data were integrated to our results and we identified 328 common relevant genes (**Figure 3**). According to their significant status of expression (+: over-expression compared to the control group; −: under-expression; 0: no change), 9 distinct meta-clusters of DEGs were defined. Four of them corresponded to genes specifically modified in DMD or in AGE. Two of them integrated differentially expressed genes in the two conditions that were anti-correlated (DMD+ AGE−; DMD− AGE+). The last three clusters included DMD and AGE correlated genes. Among all of those cross-validated genes, only 30 had a substantial sex-biased expression: 16 were over expressed in male muscle and 14 in female.

### Blind validation: data processing from a wide panel of public studies

**Figure 4:**
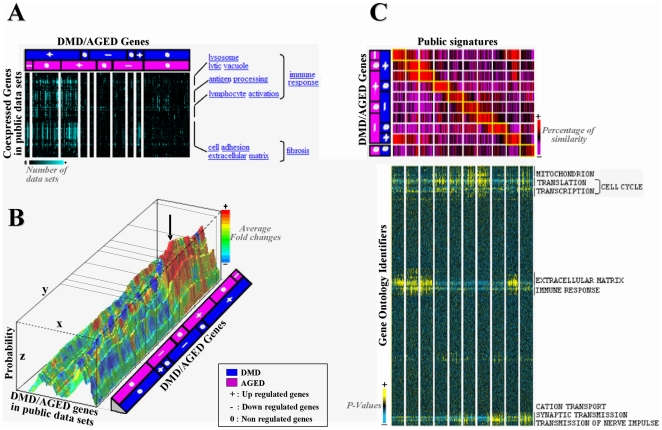
Blind validation: Meta-analysis across a wide panel of public studies. **A-** Meta-clusters of co-expressed genes: hierarchical clustering was applied to genes commonly extracted from our DMD/AGED meta-clusters in one hand (column) and 1,471 **Gemma microarray** data sets on the other hand (line). Each cell of the matrix corresponds to the number of data sets were a gene is found significantly co-expressed to the other. Light blue is attributed to high number of co-expressed genes and black to small number co-expressed genes. Functional annotation of highly co-expressed gene clusters was performed (right hand). Genes associated to immune response and fibrosis were found to be co-expressed in our DMD/AGED meta-clusters and a high number of Gemma data sets. **B-** Meta-clusters of data sets having similar significant fold-changes: hierarchical clustering was applied to fold changes values extracted from our DMD/AGED meta-cluster in one hand (y axis) and 1,515 data sets extracted from **GeneChaser** on the other hand (x axis). Each point of the x;y matrix corresponds a significant fold change value. Each fold change value is associated to a rainbow color palette: fold change values upper than 1 are with hot colors and fold values lower than 1 are with cold colors. z axis corresponds to probability associated to this fold-change to be true. The arrow delimits a cluster of studies associated to invasion processes where relevant similar fold changes are observed. **C-** Top panel: heat map presenting overlapping similar signatures: gene composition of our 10 DMD/AGE meta-clusters ( = signatures) (horizontally), is compared to ∼20,000 public transcriptional signatures (vertically). Those 20,000 signatures were extracted from ∼1,500 microarray dataset using **TBrowser**. Each cell corresponds to a percentage of similarity in gene composition between each gene list. A color code is attributed to each cell: red for good percentage of similarity and purple for bad values. For each meta-cluster, the 50 top overlapping clusters from the database were retained. Bottom panel: horizontal hierarchical clustering of best hit public signatures (columns) on the basis of their enrichment p-value found in Gene Ontology (row). A color code is attributed to each p-value (cell): yellow<black<blue. Some public signatures extracted from comparison to our DMD/AGE signatures all gather in same GO annotated functions: mitochondrion, cell cycle, extra cellular matrix, immune response, and nerve transmission.

We interrogated large compendia of re-analyzed public microarray datasets to assess the reliability of our results. We identified common transcriptional studies using different meta-analysis approaches (**Figure 4**). These analyses enabled us to re-validate, the meta-clusters in term of coexpression (**Figure 4.A**), significant fold changes (**Figure 4.B**) and gene signatures similarities (**Figure 4.C**). This validation was done with a high level of confidence (p-value<0.05), across a wide range of conditions (from normal to pathological), in various species. We identified for each of the meta-gene (obtained from our orientated meta-analysis) recurrent co-expression neighbors across multiple datasets (**Figure 4.A**). The results are exemplified for the meta-genes over-expressed in DMD, and DMD+AGE muscle. We found a clear and specific association with genes implicated in immune response and fibrosis. We looked for datasets sharing our differentially expressed meta-genes (**Figure 4.B**). These studies mainly involved up-regulated genes in invasion processes: immune response and regulation, cell adhesion to ECM components and ability to remodeling the extra-cellular space (See **Table S2** for some particular examples).

In the third strategy (**Figure 4.C**), the composition of several gene expression signatures was compared to our meta-clusters. The meta-cluster of genes over-expressed in DMD and DMD+AGE were similar to gene signatures involved in the ‘ECM’ and the ‘immune response’. Genes under-expressed both in DMD and AGE group (DMD− AGE−) shared high degree of similarity with those playing a role in mitochondrial function. Similarities between gene signatures involved in cell cycle and our meta-genes specifically up-regulated in AGE or down-regulated in DMD (DMD− AGE+, DMD− AGE0, DMD− AGE−) were found. Genes specifically down regulated in AGE muscle (DMD0 AGE−) strongly resembled gene signatures implicated in the synaptic and nerve transmission.

### Co-regulation and functional reliability of meta-clusters

**Figure 5:**
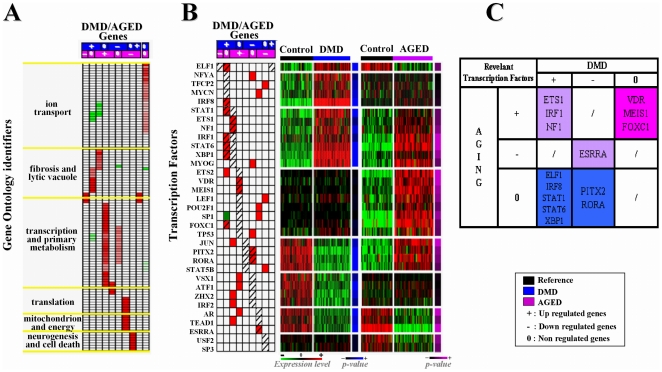
Meta-analysis of microarrays coupled with bioinformatics resources. **A-** Functional Annotation: GO term enriched subgroups (Line) of each of our DMD/AGE meta-cluster (columns) were defined via GoMiner. Significant enrichment or impoverishment in GO terms (at 5% risk) was shown respectively with red and green colors. The intensity of the color is directly proportional to the enrichment score. Each GO term enriched subgroup was clusterized together on the basis of the generic function they belong to. Six main function were identified, each contributing significant information to the overall interpretation of results. **B-** Transcription factors associated with DMD/AGE meta-clusters. Left panel: significant enrichment values (cells) in transcription factor binding sites (rows) were attributed to each of our meta-cluster (column) mining transcription factor (TF) data of MSignDB from GSEA. Red and green colors were respectively attributed to significant enrichment or impoverishment value in transcription factor binding sites (TFBS) at 5% risk (Fisher's test). Hatched squares indicate expected enrichment according to the expression level of the corresponding transcription factor (TF). Right panel: for each transcription factor (rows), expression values (cells) extracted from our DMD/AGE meta-clusters (columns) was reported. Red was applied to high level expression values, black to middle expression values and green to low expression values. High p-values obtained from T-test are visualized with blue (DMD group) or pink AGE group. Transcription factors for which enrichment values were significant in one hand and for which expression values were differential on the other hand were selected. **C-.** Fourteen significantly over expressed Transcription factors with significant enrichment of TFBS in the promoters of genes in at least one of our meta-cluster were identified. ELF1, IRF8, STAT1, STAT6 and XBP1 were associated with the meta-cluster DMD+; ETS1, IRF1 and NF1 with DMD+ AGE+; FOXC1, VDR and MEIS1 with AGE+; PITX2 and RORA with DMD−; and ESRRA with DMD− AGE−.

We searched for GO terms significantly biased (either enriched or depleted) in the different meta-clusters compared to the overall GO terms found (**Figure 5.A**). Functional annotation corroborates results inferred from the blinded meta-analyses (see **Figure S1** available on line). Remarkably, invariant genes (DMD0 AGE0) were found to support enrichment of GO terms representative of ‘ion transport’. Most of these GO terms were even under-represented in up-regulated genes (DMD+ and AGE+). A ‘fibrosis’ and ‘lytic vacuole’ scheme was specifically associated with genes over-expressed in DMD muscle (DMD+), including those common with AGE (DMD+ AGE+). Many of these GO terms were under-represented among down-regulated genes in DMD muscle. In this last group (DMD−), ‘translation’ with ‘mitochondrion’ and ‘energy’ processes were found to be enriched and shared with the group of down regulated genes in AGE. Finally, while GO terms involved in ‘primary metabolism’ and ‘transcription’ were specifically enriched in DMD down or AGE up regulated genes, a specific over-representation of genes involved in “neurogenesis” and “cell death” was detected in down-regulated genes in AGE process.

Benefiting from the results of the orientated meta-analysis, we also searched for transcription factors (TF) differentially expressed in at least DMD or AGE. Enriched binding sites of 14 over expressed Transcription factors were found in the promoter of genes identified in at least one of our meta-cluster (**Figures 5.B and 5.C**)

## Discussion

### Identifying robust meta-signatures of pathophysiological muscle

Progressive pathophysiology is a complex process involving many pathways downstream of the primary molecular insult. Microarray analyses provide comprehensive quantitative assays for transcripts and have been broadly applied to assess alterations of gene expression in diseases including neuromuscular disorders [16], [17]. In this study, we found 528 differentially expressed genes in at least DMD or AGE group comparing to a reference group of young and healthy subjects. Expression of 10 of those genes was checked by quantitative RT-PCR analysis for 3 new subjects in each DMD and AGE group. This approach has proved to be successful to validate microarray data [15]. However, microarrays are extremely sensitive to noise from experimental variables as sample size [18]. This, combined with improper analysis or validation, may leave some microarray-based studies to results that are not robust [19]–[22]. Cross-validation studies (meta-analysis) needs to be assessed across platforms and species to validate gene changes that would often be difficult to distinguish in single datasets [23]–[25]. Moreover in skeletal muscle, important variations in gene expression have been described considering sex type and muscle allotypes among disease status [26].

We have performed a re-analysis and meta-analysis of 12 publicly available microarray datasets related to DMD or AGE studies to comfort our initial data. The results enabled us to cross-validate 328 gene expression profiles in at least one of the two DMD/AGE examined situations. We reinforced the molecular signature of muscle atrophy [27] by identifying similar deregulations in various physiological situations (co-expression neighbors, differential fold changes and gene signatures). While the meta-analyzed public studies included limb muscles (most often quadriceps femoris), our study explored fascia lata tensor and paravertebral muscles. Thus cross-validated gene expressions we proposed were likely to be independent from a muscle allotype effect. According to their expression status in the two situations, the cross-validated genes could be classified into 9 distinct gene signatures (or meta-clusters). Among these genes, we could only identify 30 genes (∼9% of the cross-validated genes) having a substantial sex-biased expression. However, in the 12 studies included in the meta-analysis, AGING and DMD effects were studied by comparison with a CONTROL population constituted of a mix of male and female samples (global proportion ∼60% male and 40% female). These comparisons may thus favor a real pathological effect (DMD or AGING) on the observed gene changes. For instance, 16 of the 30 genes had a higher expression in male muscle. Nine of the 16 genes were in fact down regulated in DMD and 8 of these 9 genes were also commonly down regulated in AGING. Despite this tendency, we argue that observed variations are mainly due to the physiological status of the muscle rather than sex influence. Sex may only influence the expression of a minor part of them (∼9%) in an additive tone.

We proved that clusters of coexpressed genes are functionally related and that tissue remodeling borrowed common pathways in DMD and AGE muscle. Indeed, two independent pioneering works identified a subset of genes that were commonly up- or down-regulated [28], [29]. Since all the diseases used for the experiments of microarrays (i.e., diabetes, cancer cachexia, chronic renal failure, fasting, and denervation) had muscle atrophy in common, the up or down genes were believed to regulate the loss of muscle components and were called ‘atrophy-related genes’ or ‘atrogenes’ [30]. Integration of gene expression profiles from several studies enabled us to highlight genes that were consistently over- or underexpressed in the two situations (DMD+ AGE+ and DMD− AGE−), thus representing meta-signatures of atrophying muscle.

### Common Gene signatures to DMD and AGE

Different causes of skeletal muscle atrophy could share a common program of changes in gene expression [31]. In our study, a group of genes involved in mitochondrial function and protein synthesis shared reduced mRNA levels in skeletal muscle from DMD and AGE groups (DMD− AGED−). Calcium dyshomeostasis, increased reactive oxygen species and mitochondrial dysfunction could play a key role in the process of the muscle fiber degeneration by activating specific pathways commonly in these two conditions. In sarcopenia, aged skeletal muscle contains a large number of muscle fibers with segmental cytochrome *c* oxidase deficiency and mitochondrial proliferation indicative of mitochondrial metabolism dysfunction [32]. Clonal expansion of mitochondrial DNA (mtDNA) mutations causes these focal muscle fiber mitochondrial alterations [33]. Apoptosis has been detected in human aged skeletal muscle and was co-localized with abnormal mitochondrial proliferation [34]. Thus, mitochondrial dysfunction, apoptosis and muscle fiber atrophy was observed in skeletal muscle of mtDNA mutator mice [35]. This could corroborate a link between mitochondria decline and protein synthesis decline observed in our study for the age group. Down-regulation of these genes may thus indicate a ‘metabolic crisis’ reflected by the chronic decline in muscle function and homeostasis. Indeed, several metabolic adaptations occur in atrophying muscles and interestingly, in many forms of muscle wasting. And expression of a variety of genes involved in glycolysis and oxidative phosphorylation are also coordinately suppressed [31].

Genes over-expressed in the two conditions (DMD+ AGED+) belonged to the cell adhesion/extracellular matrix family, and those encoding components of the immune response. Those effects could be observed in other pathologies (e.g. spastic paraplegia) but also in various tissues including muscle as an effect of AGE. Recently, we demonstrated that in advanced heart failure, up and down-regulated genes in the deteriorating samples involved proteins of the extracellular matrix and mitochondrion, respectively [36]. The extracellular matrix molecules are critical for creating the cellular environment required during muscle development and morphogenesis and muscle regeneration is a highly efficient and reproducible process [37]. In senescent and DMD muscles, tissue fibrosis and disruption of regenerative potential are observed. This results in further perturbation of skeletal muscle and activated immune cell infiltration [38]–[40].

While in DMD inflammation and fibrosis is an important characteristic that for some researcher is the main causes of the muscle loss, inflammation in skeletal muscle aging has long been a debated point. There is a growing body of evidence suggesting that sarcopenia is a smoldering inflammatory state driven by cytokines and oxidative stress since elevated levels of interleukin-6 and C-reactive protein are often detected (see “Inflammation and AGING” in **Data S1**). In our study, 33% of the genes (25% of the total) that could be linked to an immune/defence response (e.g. IRF1, ETS1, STAT6, IL4R,…) as determined by database searches were found in various published inflammatory signatures (see part 2 of **Table S1** for a description of the Immune related genes), including the one occurring in the aging skeletal muscle from rhesus monkeys [41]. In this study, while down-regulated genes mainly involved proteins of the energy metabolism, up-regulated genes encoded proteins linked to an inflammatory/immune response, representing the largest class of transcripts that display a large (2-fold or more) change in expression pattern with aging. These finding thus suggests that as found in the DMD, a cytokine-mediated inflammatory response also occurs in the AGING muscle which could be involved in the atrophying process.

### Identifying Common deregulated pathways

We found that down regulation of genes in both conditions (DMD− AGED−) may be mediated by the orphan nuclear estrogen-related receptor α (ESRRA), a recently discovered regulator of mitochondrial biogenesis [42]. Bioinformatic analyses revealed that it belongs to the mitochondrial co-expression module in the mammalian skeletal muscle [43]. Moreover, contractile and energy generation promoters also contain the ESRRA motif in co-expression modules deduced from a wide range of degenerative heart disease [44]. ESRRA was recently demonstrated to target a common set of promoters involved in the uptake and production of energy substrate across the mitochondrial membranes, intracellular fuel sensing (Ca2+ handling and contractile work…) [45]. Association of down-regulation of ESRRA to muscle fiber loss in DMD and with AGE as a direct or a secondary consequence still remains to be elucidated.

In DMD+ AGED+, overexpression of several genes is related for the secondary response composed of the connective tissue infiltration and extracellular matrix proliferation. Our analysis identified three transcription factors –the avian v-ets erythroblastosis virus E26 oncogene homolog 1 (EST1), the interferon regulatory factor 1 (IRF1) and the neurofibromin1 (NF1)-related to this response. They may coordinate gene changes in the invading cells to regulate the immune response and/or the extracellular matrix remodeling. Indeed, the proto-oncogene ETS1 is involved in both normal and pathological functions and is expressed in a variety of cells, including endothelial cells, vascular smooth muscle cells and epithelial cells. It regulates the expression of several angiogenic and extracellular matrix remodeling factors promoting an invasive phenotype [46]. The high expression of ETS1 observed in our conditions could be correlated to infiltrating activated T-cell [47]. Thus ETS1 would be a key mediator of the extracellular-signal induced activation. IRF1 is a multifunctional transcription factor acting downstream of the JAK/STAT pathway, and notably involved in cell growth regulation, inflammation, immune response and immune activation [48]. It belongs to the immune transcriptional module in the muscle [43].

NF1 is a tumor suppressor protein encoded by the *NF1* gene. Mutations in the *NF1* gene cause Neurofibromatosis 1 which is mainly characterized by the development of multiple benign tumors of nerves and skin. The musculoskeletal system is also often affected (e.g. scoliosis and bowing of the lower legs). NF1 is predominantly detected in neurons, nonmyelinating Schwann cells and oligodendrocytes but is also expressed in skeletal muscle or smooth muscle [49]. Indeed muscle-specific isoforms of NF1 have been identified [50]. NF1 is required for the development and the maintenance of the musculoskeletal system and could contribute to the musculoskeletal problems in patients affected with neurofibromatosis type 1. As shown in our study, NF1 is involved in the rearrangement of cytoskeletal components. Skeletal muscle from mice with NF1 inactivation gene shows a dystrophic skeletal muscle aspect with fibrosis and reduced number of muscle fibers and altered differentiation of satellite cells [51].

### Specific Gene signatures to DMD or AGED

Finally, our results show that in both situations, different causes may lead to the same consequences as observed by common gene signatures of both DMD and AGED muscle. However, according to the other gene signatures observed in one of the two situations, specific pathways also exist and will be the scope of next studies to identify the precise role of transcription factors (e.g. ESRRA, MYOG, and VDR) as we recently developed for other tissues [52]. For instance, while in the DMD muscle the lack of dystrophin is clearly identified as the primary cause of muscle loss, in AGED the process has not been elucidated yet. However, we found that genes down-regulated only in the AGED muscle (DMD0 AGED−) suggests a motor unit remodeling process as hypothetical primary cause. Indeed, several evidences suggest that one principal mechanism accounting for the age-related muscle mass loss could be the age-related decline of peripheral nervous system. Motor units and neuromuscular junction demonstrate anatomic and physiological remodeling with age such as reduced total number, relative increase of muscle fibers per motor unit, increase in pre-synaptic nerve terminal branching associated with increased quanta content [53]. The continual loss of fast motor neurons would lead to a progressive process of myofiber denervation associated with incomplete re-innervation by slow motor neurons contributing to the ineluctable muscle atrophy with a denervation rate probably exceeding the re-innervation rate in the elderly. Indeed, remodeling of the neuromuscular junction precedes sarcopenia related alterations in myofibers [54]. These findings are now under new investigations to clarify the precise role of these biomarkers - particularly the transcription factors- in the context of muscle pathology.

To conclude, if the number of relevant studies related to muscle continues to rapidly increase, efficient exploitation of these wide data is frustrated by the lack of an integrated data mining platform or other unifying bioinformatic resource. To facilitate the integrative analysis of microarray data on muscle, it could be thus useful to develop automatic tool relying on meta-analysis approaches (coexpression, signature, fold change). This will allow to focus on mechanisms involved in regulating the observed phenotypic changes. Indeed, by exploring common regulatory networks, we will identify key regulators of muscular tissue remodeling.

## Materials and Methods

### In laboratory study

#### Patient samples

Written informed consents were collected for all the patients involved in our study that was approved by the ethical committee of the Nantes University Hospital (France). Muscle tissue samples (paravertebral or fascia lata muscles) were rendered anonymous before beginning the study (see Table 1 for a complete description). They were from four male patients (ages: 11–13 years) undergoing orthopedic surgery. Patients were diagnosed with DMD (DMD group) on the basis of clinical examination, histological analysis and absence of dystrophin at the immuno-fluorescence analysis. Four AGE samples were collected in subjects (ages: 42–65 years; 1 male and 3 females) during surgery for idiopathic scoliosis (AGE group). Reference skeletal muscle samples (CONTROL group) were obtained from seven subjects (ages: 14–30 years; 2 males and 5 females) undergoing surgery for idiopathic scoliosis.

**Table 1:** Clinical features of the three groups of patients.

	Name	Sex	Age	Clinical Examination	Muscle	Treatment
**DMD Group**						
	DMD1	♂	12	Armchair since 1 year, minor cognitive impairment	Fascia lata tensor	No
	DMD2	♂	11	Armchair since 2 years	Fascia lata tensor	No
	DMD3	♂	11	Armchair since 4 years	Fascia lata tensor	No
	DMD4	♂	13	Armchair since 2 years	Paravertebral	No
**AGE Group**						
	AGING1	♂	42	Idiopathic scoliosis	Paravertebral	No
	AGING2	♀	65	Idiopathic scoliosis	Paravertebral	No
	AGING3	♀	59	Idiopathic scoliosis	Paravertebral	No
	AGING4	♀	47	Idiopathic scoliosis	Paravertebral	No
**CONTROL Group**						
	CONTROL1	♀	30	Idiopathic scoliosis	Paravertebral	No
	CONTROL2	♀	19	Idiopathic scoliosis	Paravertebral	No
	CONTROL3	♂	14	Idiopathic scoliosis	Paravertebral	No
	CONTROL4	♀	16	Idiopathic scoliosis	Paravertebral	No
	CONTROL5	♀	27	Idiopathic scoliosis	Paravertebral	No
	CONTROL6	♀	16	Idiopathic scoliosis	Paravertebral	No
	CONTROL7	♂	18	Idiopathic scoliosis	Paravertebral	No

#### Microarray experiments and cluster analysis

Microarray preparation and hybridization, expression data acquisition and processing were described in **Data S1**. The transcriptome experiment was performed using the Nantes' Integrative Genomic Platform (see **Table S3** for a complete description) and a protocol previously published (see for details [36], [55]). Data were normalized and analyzed using the Micro-Array Data Suite of Computed Analysis (MADSCAN) [56].

A total of 2,745 genes were found to be consistently expressed in our experimental conditions. These gene expression profiles were used to classify genes and biological samples using a hierarchical clustering method with the Gene Cluster program. The input matrix consisted of the log_2_-transformed median of the replicate expression values for each gene. The clustering method employed was an average linkage with the Pearson correlation coefficient as a similarity metric. Results were displayed using the Java Treeview. Our dataset has been deposited in the GEO database (Series record GSE32720).

#### Real time RT-PCR

Quantitative RT-PCR (Taqman Gene expression assays, Applied Biosystems) was used to assess the reliability of the measures obtained from microarray experiments. A set of 10 genes distributed along the entire range of gene expression variation for DMD and AGE subjects was selected. Helicase with zinc finger domain (HELZ) was taken as an internal standard. PCR reactions were performed on the same samples that those used for the microarray experiments. Three of the four DMD and three of the four AGE subjects were compared individually to the pool of seven control subjects (CONTROL). Duplicate experiments were performed and each experiment contained duplicate PCR reactions. Mean gene expression ratios were calculated for each DMD and AGE subjects versus CONTROL.

#### Statistical analysis

Differentially expressed genes (DEGs) in DMD or AGE groups compared to control group were detected using two statistical methods specifically adapted to microarray data: SAM (Significance Analysis of Microarrays) and LIMMA (Linear Models for MicroArray data). To minimize false positive cases (false discovery rate, FDR), genes selected only by both methods were admitted in the final list of DEGs (FDR<0.02; p<0.01). A hierarchical clustering was also applied on clusters of DEGs.

### Orientated validation: data processing from independent DMD and AGE public studies

Microarray datasets and related information on samples were downloaded from GEO at the National Center for Biotechnology Information (NCBI, Bethesda, MD). They include 6 series related to DMD [GSE6011, GSE1007, GSE1004, GSE3307, GSE465, GSE1764] and 6 series related to AGE [GSE1428, GSE10760, GSE80, GSE8479 and GSE9676 gathering GSE362 and GSE674]. All these data sets (raw, normalized and analyzed data) can also be retrieved in the MADMuscle database (www.madtools.org, [57]), a resource dedicated to the analysis of muscle transcriptome data. Normalization: for each individual data set, non-linear effects such as background or saturation were corrected by LOWESS as previously described [15].

For each GEO platform (GPL), information on probe-sets was gathered in the MADGene database (www.madtools.org) and completed with related information collected from the NCBI Entrez gene database and Unigene databases [58], [59]. Based on this annotation, genes found as differentially expressed in our study were retrieved in each independent GEO dataset.

Each dataset was individually preprocessed applying a logarithmic transformation and a median centering on genes [60], so that relative variations rather than absolute values were used for interpretation. When multiple probes were found for a same gene, only the probe with the smallest p-value (student's T test) between distinguished experimental groups (DMD *vs* CONTROL or AGE *vs* CONTROL) was kept for further analyses [61]. The data were merged into two independent meta-matrixes dedicated to DMD and AGE effects respectively. In each meta-matrix, a Student's T test (p-value<0.01) was applied on each gene expression meta-profile to identify significant variations between the studied groups. Additionally, genes with sex biased expression values were identified using the same strategy. Significant results were ranged according to their validation status (over-expression, under-expression or no change compared to CONTROL) and displayed with Java Treeview.

### Blind validation: data processing from a wide panel of public studies

DMD/AGE isolated genes from previous Orientated Meta-analysis were validated in terms of co-expression, fold-changes and gene signatures exploring thousands of data expression in various conditions (pathological or not) and species (including human).

#### Co-expression analysis

Reliable co-expression neighbors of DMD/AGE genes were found across multiple independent datasets using GEMMA on the basis of their expression profiles correlation [62], [63].

#### Differential expression analysis

We identified GEO public datasets in which significant expression variations were observed using GeneChaser server [64].

#### Gene signatures

For each DMD/AGED validated cluster of DEG, the 50 significant overlapping gene sets were sought in a large collection of transcriptional signatures using TBrowser [65].

### Functional annotation and regulatory mechanism identification

Functional annotation was performed using GoMiner tool and gene ontology (GO) classification scheme. Regulatory mechanisms were identified mining transcription factor (TF) data of MSignDB from GSEA [66]. For each analyzed set of genes (DMD/AGE), significant bias (over or under-representation) of GO identifiers and transcription factor binding sites (TFBS) were calculated using the Fisher's exact test and the entire set of 2,745 expressed genes as the reference population. Only bias with p-value lower than 0.05 were considered for interpretation.

## Supporting Information

Figure S1 **Functional analysis of the meta-clusters.** The functional analysis was performed for the three domains covered by the ontology: “Cellular component” (left upper panel) which corresponds to the parts of a cell or its extracellular environment; “molecular function” (left lower panel) which includes the elemental activities of a gene product at the molecular level; “biological process” corresponding to the operations or sets of molecular events with a defined beginning and end, pertinent to the functioning of integrated living units (cells, tissues, organs, and organisms). For each domain, Gene Ontology (GO in lines) profile analysis of the meta-cluster (set of cross validated genes in columns) is done. Bias of GO terms are depicted as colored squares (green for under-represented terms, and red for over-represented terms), with color intensity directly reflecting the range of the bias and corresponding to the log10 of the GO enrichment scores. The terms significantly (at 5% risk) associated with any of the 6 meta-clusters are denoted by a white star.
(TIF)

Table S1 **Genes differentially expressed in DMD and AGING patients.** Part 1: List of all the genes noted on GO biological process and differentially expressed in DMD and AGING patients. Part2: List of the Immune/Defense/Inflammation related genes differentially expressed in DMD and AGING patients.
(XLS)

Table S2 **Examples of studies identified as having similar gene over-expression as observed in DMD muscle.**
(XLS)

Table S3 **The 3588 genes spotted on the microarrays.**
(XLS)

Data S1 **Methods.**
(PDF)
