# Supplementary figures and images for: Immune Response and Mitochondrial Metabolism Are Commonly Deregulated in DMD and Aging Skeletal Muscle
Source: PLoS One. 2011 Nov 9;6(11):e26952. doi: 10.1371/journal.pone.0026952 (PMC3212519; doi:10.1371/journal.pone.0026952)

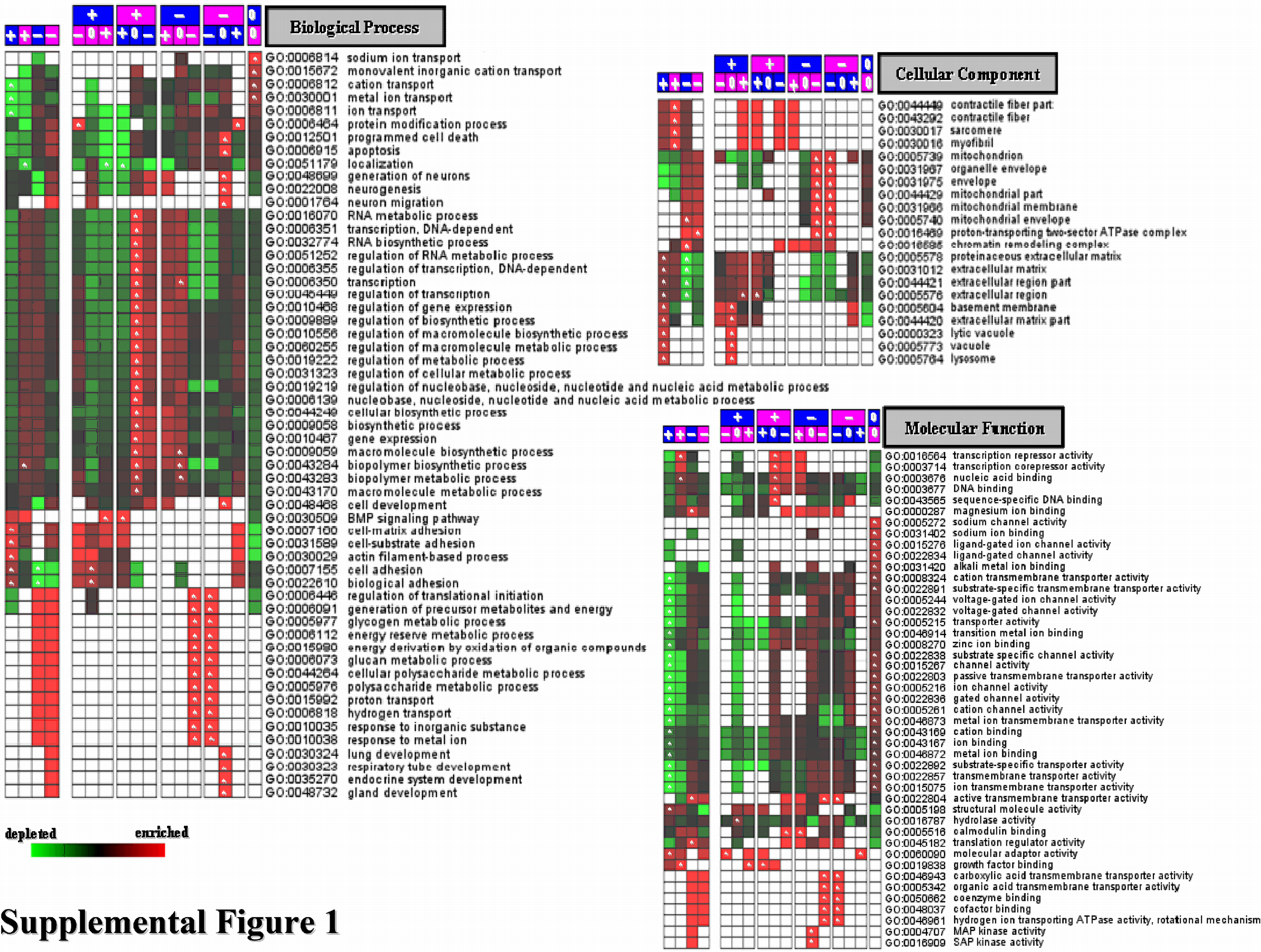

Supplement: Figure S1 — Functional analysis of the meta-clusters. The functional analysis was performed for the three domains covered by the ontology: “Cellular component” (left upper panel) which corresponds to the parts of a cell or its extracellular environment; “molecular function” (left lower panel) which includes the elemental activities of a gene product at the molecular level; “biological process” corresponding to the operations or sets of molecular events with a defined beginning and end, pertinent to the functioning of integrated living units (cells, tissues, organs, and organisms). For each domain, Gene Ontology (GO in lines) profile analysis of the meta-cluster (set of cross validated genes in columns) is done. Bias of GO terms are depicted as colored squares (green for under-represented terms, and red for over-represented terms), with color intensity directly reflecting the range of the bias and corresponding to the log10 of the GO enrichment scores. The terms significantly (at 5% risk) associated with any of the 6 meta-clusters are denoted by a white star. (TIF) [file pone.0026952.s001.tif]
