# Supplementary material for: Immune Response and Mitochondrial Metabolism Are Commonly Deregulated in DMD and Aging Skeletal Muscle
Source: PLoS One. 2011 Nov 9;6(11):e26952. doi: 10.1371/journal.pone.0026952 (PMC3212519; doi:10.1371/journal.pone.0026952)
Supplement: Data S1 — Methods. (PDF) [file pone.0026952.s005.pdf]

Supplemental data

# methods

Study design

Each of the 8 biological samples was compared to a common reference sample consisting of a pool of equal quantities of mRNA from 7 other biological samples. This complex mRNA pool was used as a standard or a common point of measurement that enabled a comparison between the individual mRNA samples.

Control of technical and biological noises inherent to microarray experiments was incorporated in the study design. To account for the technical fluctuation of the expression measurements, eight technical replicate values were obtained for each muscle sample. To determine the fluctuation due to tissue sampling, two biological replicate samples were obtained from each biopsy.

"Myochips": Microarray production

The 3588 genes represented on the microarray were selected for involvement in skeletal muscle. Selection was based on (i) subtractive hybridization experiments using a rat model of muscular atrophy and *mdx* mice [1,2], (ii) genome-wide microarray hybridizations in two muscular pathologies: Duchenne Muscular Dystrophy and Myotonic Dystrophy type 1 (unpublished data). Other genes involved in muscular disease were added after literature database searches. The supplemental table 3 shows the 3588 genes spotted on the Myochips. Microarrays were prepared in-house using 50-mer oligonucleotides probes (MWG Biotech). The probes were spotted onto epoxy-silane coated glass slides using the Lucidea Array Spotter (AMERSHAM, Uppsala, Sweden). Each gene was spotted in quadruplicate and therefore spatially distributed on the slide. For more information see: http://cardioserve.nantes.inserm.fr/ptf-puce/myochips_en.php

Target preparation

Normal and patient total RNA was purified using Trizol Reagent (Life technologies). A small aliquot of RNA was then used for quantification and quality control using the RNA 6000 labchip kit and Agilent Bioanalyser (Agilent Technologies, Palo Alto, United States). Poly (A)+ RNA was purified from total RNA using oligo-dt linked oligotex resin (QIAGEN). Cy3- and Cy5-labeled cDNA was prepared using the CyScribe cDNA post labeling Kit (Amersham Pharmacia Biotech). Samples from DMD and AGING patients were each labeled individually with Cy5. Reference patients were pooled and then labeled with Cy3.

Hybridization

Microarrays were prehybridized for 1 hour at 42° C (in 10% BSA, 20XSSC, 10% SDS). 0.5 µg of labeled sample cDNA was mixed with 0.5 µg of labeled reference cDNA in 40 µl of hybridization mix (50X denhardt, 20XSSC, 10 µg/µl PolyA RNA, 10 µg/µl Yeast tRNA, 10% SDS, 50% formamide) and hybridized to the microarrays. Microarray hybridization was carried out overnight at 42° C in a humidified chamber. Finally, slides were washed, dried by centrifugation and read by a scanner. Two independent hybridizations were performed for each DMD and AGING patient sample.

Image acquisition and data consolidation

Array scanning was carried out using a GSI lumonics LITE dual confocal laser scanner with ScanArray Microarray Analysis Software. Raw scanner images were analyzed with Genepix Software (AXON LABORATORY). Analysis of the expression level was performed using MADSCAN (MicroArray Data Suites of Computed Analysis) developed in our laboratory [3]. MADSCAN can be applied to one microarray or a batch of replicated microarrays and consists of:

- *Physical validation,* aiming to flag flawed spots and to extract information from borderline features in raw data matrices.

- *Normalization*, in order to minimize experimental systematic biases so that the observed variation arises from biological differences rather than from defects in the microarray technology. Invariant genes were selected using the rank invariant method [4]. A non-linear regression method (lowess fitness) was applied to the invariant genes to calculate the normalization correction, which was then used to normalize all spots on the array [5].

- *filtering* to remove genes of which all signal intensities were below the background level.

- *Scaling* to bring the internal variance within or between slides within the same range.

- *Outliers:* the filtered and normalized quantification data matrices must be compared to statistically validate the replicates and to remove outliers. An outlier is a suspect value in terms of its relative distance to the median value. We labeled outliers with the MAD test (Median absolute deviation test), which is a modified z-test where the median absolute deviation is used instead of the standard deviation in the z score calculation.

# Inflammation and AGING

Acute inflammation is thought to play an important role in muscle repair [6] but there is a growing body of evidence favouring the fact that chronic inflammation may also be associated with the loss of muscle mass (sarcopenia) and muscle strength with aging [7]. In this situation, sarcopenia is though to be a smoldering inflammatory state driven by cytokines and oxidative stress [8].

In the aging skeletal muscle from rhesus monkeys [9], while down-regulated genes mainly involved proteins of the energy metabolism, up-regulated genes encoded proteins linked to an inflammatory/immune response along with stress response genes. The genes from the inflammation signature represented the largest class of transcripts that display a large (2-fold

or more) change in expression pattern with aging. Higher levels of pro-inflammatory cytokines (interleukin-6 (IL6), tumor necrosis factor-α (TNF)) and C-reactive protein (CRP) increase the risk of muscle strength loss [10] and are correlated with lower muscle mass in healthy older persons [11], even if the increase is moderate. Increased expression of IL6 has implicated cytokine-mediated events in reduced skeletal muscle function with age [12,13] although IL6 may also possess anti-inflammatory properties within this context [14].

Aging results in a rise in basal inflammation that is thought to contribute to sarcopenia by causing a decline of physical functioning through the catabolic effects of inflammatory markers on muscle [15,16]. Such stimulation of protein catabolism, probably through the increased activity of the Ubiquitin/Proteasome proteolytic pathway, has been shown for instance in rat were administration of IL6 or TNF causes muscle protein breakdown [17,18].

Reference List

1. Cros N, Tkatchenko AV, Pisani DF, Leclerc L, Leger JJ, Marini JF, Dechesne CA (2001) Analysis of altered gene expression in rat soleus muscle atrophied by disuse. J Cell Biochem 83: 508-519.

2. Tkatchenko AV, Le CG, Leger JJ, Dechesne CA (2000) Large-scale analysis of differential gene expression in the hindlimb muscles and diaphragm of mdx mouse. Biochim Biophys Acta 1500: 17-30.

3. Le MN, Lamirault G, Bihouee A, Steenman M, Bedrine-Ferran H, Teusan R, Ramstein G, Leger JJ (2004) A dynamic, web-accessible resource to process raw microarray scan data into consolidated gene expression values: importance of replication. Nucleic Acids Res 32: 5349-5358.

4. Tseng GC, Oh MK, Rohlin L, Liao JC, Wong WH (2001) Issues in cDNA microarray analysis: quality filtering, channel normalization, models of variations and assessment of gene effects. Nucleic Acids Res 29: 2549-2557.

5. Yang YH, Dudoit S, Luu P, Lin DM, Peng V, Ngai J, Speed TP (2002) Normalization for cDNA microarray data: a robust composite method addressing single and multiple slide systematic variation. Nucl Acids Res 30: e15.

6. Tidball JG (2005) Inflammatory processes in muscle injury and repair. Am J Physiol Regul Integr Comp Physiol 288: R345-R353.

7. Roth SM, Metter EJ, Ling S, Ferrucci L (2006) Inflammatory factors in age-related muscle wasting. Curr Opin Rheumatol 18: 625-630.

8. Meng SJ, Yu LJ (2010) Oxidative stress, molecular inflammation and sarcopenia. Int J Mol Sci 11: 1509-1526.

9. Kayo T, Allison DB, Weindruch R, Prolla TA (2001) Influences of aging and caloric restriction on the transcriptional profile of skeletal muscle from rhesus monkeys. Proc Natl Acad Sci U S A 98: 5093-5098.

10. Schaap LA, Pluijm SM, Deeg DJ, Visser M (2006) Inflammatory markers and loss of muscle mass (sarcopenia) and strength. Am J Med 119: 526-17.

11. Visser M, Pahor M, Taaffe DR, Goodpaster BH, Simonsick EM, Newman AB, Nevitt M, Harris TB (2002) Relationship of interleukin-6 and tumor necrosis factor-alpha with muscle mass and muscle strength in elderly men and women: the Health ABC Study. J Gerontol A Biol Sci Med Sci 57: M326-M332.

12. Barbieri M, Ferrucci L, Ragno E, Corsi A, Bandinelli S, Bonafe M, Olivieri F, Giovagnetti S, Franceschi C, Guralnik JM, Paolisso G (2003) Chronic inflammation and the effect of IGF-I on muscle strength and power in older persons. Am J Physiol Endocrinol Metab 284: E481-E487.

13. Ferrucci L, Harris TB, Guralnik JM, Tracy RP, Corti MC, Cohen HJ, Penninx B, Pahor M, Wallace R, Havlik RJ (1999) Serum IL-6 level and the development of disability in older persons. J Am Geriatr Soc 47: 639-646.

14. Tilg H, Dinarello CA, Mier JW (1997) IL-6 and APPs: anti-inflammatory and immunosuppressive mediators. Immunol Today 18: 428-432.

15. Ferrucci L, Penninx BW, Volpato S, Harris TB, Bandeen-Roche K, Balfour J, Leveille SG, Fried LP, Md JM (2002) Change in muscle strength explains accelerated decline of physical function in older women with high interleukin-6 serum levels. J Am Geriatr Soc 50: 1947-1954.

16. Roubenoff R (2007) Physical activity, inflammation, and muscle loss. Nutr Rev 65: S208-S212.

17. Goodman MN (1991) Tumor necrosis factor induces skeletal muscle protein breakdown in rats. Am J Physiol 260: E727-E730.

18. Goodman MN (1994) Interleukin-6 induces skeletal muscle protein breakdown in rats. Proc Soc Exp Biol Med 205: 182-185.
